# Complete Genome Sequence of *Pseudomonas* sp. Strain phDV1, an Isolate Capable of Efficient Degradation of Aromatic Hydrocarbons

**DOI:** 10.1128/MRA.01171-18

**Published:** 2019-01-10

**Authors:** Hao Xie, Giannis Valsamidis, Eirini Mathioudaki, Georgios Tsiotis

**Affiliations:** a Department of Molecular Membrane Biology, Max Planck Institute of Biophysics, Frankfurt am Main, Germany; b Department of Chemistry, University of Crete, Heraklion, Greece; University of Arizona

## Abstract

Pseudomonas sp. strain phDV1 is a Gram-negative bacterium capable of degrading aromatic hydrocarbons. Here, we present the complete genome sequence of this strain, which consists of 4,727,682 bp, with a 62.3% G+C content and 4,574 genes. Multiple genes responsible for the degradation of aromatics are present in this strain.

## Abstract (precis)

Pseudomonas sp. strain phDV1 is a Gram-negative bacterium capable of degrading aromatic hydrocarbons. Here, we present the complete genome sequence of this strain, which consists of 4,727,682 bp, with a 62.3% G+C content and 4,574 genes.

## ANNOUNCEMENT

Pseudomonas sp. strain phDV1 was isolated from a petroleum-contaminated site in Denmark (1, 2). Pseudomonas sp. phDV1 was shown, through gas chromatography-mass spectrometry (GC-MS) and proteomics analysis, to efficiently metabolize phenol, toluene, *o*-cresol, naphthalene, and 1,2,3-trimethylbenzene (1–4). The genome of Pseudomonas sp. phDV1 was chosen for sequencing due to its ability to degrade aromatics and grow in harsh hydrocarbon-containing environments.

A single colony of Pseudomonas sp. phDV1 was picked and cultured in lysogeny broth at 32°C. Genomic DNA was extracted from the cultures using the Gentra Puregene Yeast/Bact. kit (Qiagen, Germany). A PacBio 10-kb sequencing library was constructed using the PacBio template prep kit following the manufacturer’s protocols (Pacific Biosciences, USA). The genome of Pseudomonas sp. phDV1 was sequenced using the PacBio RS II system on single-molecule real-time (SMRT) cells using PacBio P6-C4 chemistry at Beijing Novogene Technology Co. Ltd. (China). The raw reads were filtered in SMRT Portal (version 3.2.0), with 0.75 as the minimum read quality and 500 bp as the minimum read length. The clean data (983,266,888 bp, 98,763 reads, 208× coverage) were *de novo* assembled using the Hierarchical Genome Assembly Process (HGAP) (5) in SMRT Portal (version 3.2.0) to generate one contig without gaps. The genome of Pseudomonas sp. phDV1 consists of a circular chromosome, which is 4,727,682 bp in length, with a G+C content of 62.3%. No plasmid was found in this strain. The genome annotation was performed using the NCBI Prokaryotic Genome Annotation Pipeline (6), which predicted 4,574 genes, including 4,081 coding sequences (CDSs), 411 pseudogenes, 4 copies of each of the rRNA genes (5S, 16S, and 23S), 65 tRNAs, and 5 noncoding RNAs.

For functional annotation, the predicted protein-coding sequences were searched against the Clusters of Orthologous Groups (COG) of proteins (7), Gene Ontology (GO) (8), and Kyoto Encyclopedia of Genes and Genomes (KEGG) databases (9). According to the annotation results, multiple genes involved in hydrocarbon degradation were found in the genome. Among them, the operon *dmpKLMNOPQBCDEFGHI* encoding proteins involved in phenol degradation resemble the canonical genetic organization of Pseudomonas sp. strain CF600 type (10). The *tmoABCDEF* cluster encoding a multicomponent toluene 4-monooxygenase, involved in the degradation of toluene and various cresol compounds, is located 6 kb upstream of the *dmp* operon. In addition, homogentisate 1,2-dioxygenase, 2-alkenal reductase, P450, xylene monooxygenase, protocatechuate 3,4-dioxygenase, and 4-carboxymuconolactone decarboxylase were present as well. The presence of these enzymes likely explains the aromatic degradation capacities of Pseudomonas sp. phDV1.

The 16S rRNA sequence analysis using the genomic-based 16S rRNA database (GRD; http://metasystems.riken.jp/grd/) revealed that Pseudomonas sp. phDV1 shares 99.93% and 99.41% similarity with Pseudomonas pseudoalcaligenes CECT5344 and Pseudomonas mendocina NK-01, respectively. At the genome level, the OrthoANIu algorithm was used to calculate the average nucleotide identity (ANI) value (11), and the Genome-to-Genome Distance Calculator (GGDC) was employed to estimate the *in silico* DNA-DNA hybridization (isDDH) value (12). The ANI and isDDH values between Pseudomonas sp. phDV1 and Pseudomonas pseudoalcaligenes CECT5344 were 96.52% and 70.30%, respectively, while both values were substantially lower (88.64% for ANI and 36.70% for isDDH) when Pseudomonas sp. phDV1 was compared to Pseudomonas mendocina NK-01. Based on these results, we suggest the transfer of Pseudomonas sp. phDV1 to the species Pseudomonas pseudoalcaligenes as strain phDV1.

### Data availability.

The PacBio sequencing reads have been deposited in the NCBI Sequence Read Archive (SRA) under the accession number SRR8212849. The whole-genome sequence has been deposited at GenBank under the accession number CP031606. The version described in this paper is the first version, CP031606.1.
